# Development of a HPLC-MS/MS Method to Determine the 13 Elements of *Semen Cuscutae* and Application to a Pharmacokinetic Study in Rats

**DOI:** 10.1155/2019/6518528

**Published:** 2019-11-06

**Authors:** Jiayuan Shen, Qi Jia, Xuhua Huang, Guangzhe Yao, Wenjuan Ma, Huamei Zhang, Huizi Ouyang, Jun He

**Affiliations:** ^1^First Teaching Hospital of Tianjin University of Traditional Chinese Medicine, Tianjin 300193, China; ^2^Tianjin State Key Laboratory of Modern Chinese Medicine, Tianjin University of Traditional Chinese Medicine, Tianjin 301617, China

## Abstract

This study developed a method for simultaneous determination of 13 elements of *Semen Cuscutae* (quercitrin, quercetin, hyperoside, caffeic acid, chlorogenic acid, luteolin, apigenin, kaempferol, isoquercitrin, cryptochlorogenic acid, isorhamnetin-3-*O*-glucoside, astragalin, and rutin) in rat plasma using high-performance liquid chromatography-tandem mass spectrometry (HPLC-MS/MS) in the negative MRM mode. The analytes were analyzed with CORTECS®C_18_ column (4.6 × 150 mm, 2.7 *μ*m) with mobile phases consisting of 0.1% formic acid in water (A) and acetonitrile (B). The intra- and interday precision of the target compounds were expressed as relative standard deviation (RSD) in the range of 0.5%–10.4%, and the accuracy of the target compounds was expressed as relative error (RE) not exceeding ±14.5% for all analytes. In the meantime, the extraction recovery of the target compounds in plasma samples ranged from 87.4% to 106.2% and matrix effect from 81.0% to 115.5%. The established method was successfully accomplished for the pharmacokinetic study of the analytes in rat plasma samples following oral administration of *Semen Cuscutae* extract, and the pharmacokinetic parameters of seven compounds were obtained.

## 1. Introduction

*Semen Cuscutae* (Longxuzi, Tusizi), the dry mature seed, belongs to *Cuscuta australis* R.Br. or *Cuscuta chinensis* Lam. of Convolvulaceae family [1, 2]. It was first recorded in the “Shen Nong's Herbal” as an upper grade drug [1]. As a well-known traditional Chinese medicine, *Semen Cuscutae* has numerous pharmacological functions, such as regulating the body's endocrine system, nourishing the liver and kidney, improving eyesight, and preventing miscarriage [3, 4]. It has also been reported to have neuroprotective, hepatoprotective, antioxidative, osteoblastogenic, and immunomodulatory properties and to have positive effects on chronic prostatitis [5–7]. There are many natural active ingredients in *Semen Cuscutae*, which includes flavonoids, lignans, polysaccharides, alkaloids, and other chemicals [1, 6, 8], with flavonoids and phenolic compounds being the predominantly bioactive constituents [9, 10].

Traditional Chinese medicine has been used in the treatment of diseases for thousands of years in China [11]. However, due to the complex composition of traditional Chinese medicine, its safety and effectiveness are still in doubt worldwide [10]. Qualitative and quantitative analysis of its components in traditional Chinese medicine has certain significance. In the recent years, the chemical studies on *Semen Cuscutae* have mainly focused on qualitative analysis of its major components using different analytical methods, such as HPLC, LC-MS/MS, and so on [12], but the pharmacokinetic studies of *Semen Cuscutae* were rarely reported. In the latest research, Zhang et al. [13] established the kidney-deficiency model on rats to expound the pharmacokinetic differences of six renoprotective compounds from *Semen Cuscutae* between normal and kidney-deficiency rats. The metabolism process in vivo of six flavonoids were clarified and showed prospective results in their study. However, it is insufficient to study the pharmacokinetics of only one kind of component, and other active ingredients from *Semen Cuscutae* need to be further investigated too.

As we know, study on the pharmacokinetics of traditional Chinese medicine can elucidate its material basis and explain the mechanism of drug absorption, distribution, metabolism, and excretion in vivo [14]. Therefore, in our study, we simultaneously separated and determined 13 elements from *Semen Cuscutae* extract by a selective HPLC-MS/MS method and analyzed their pharmacokinetics data. This research would contribute to the understanding of the metabolism of these elements, as well as the mechanism of action of *Semen Cuscutae*.

## 2. Material and Methods

### 2.1. Analytical Standards and Reagents

**Figure 1:**
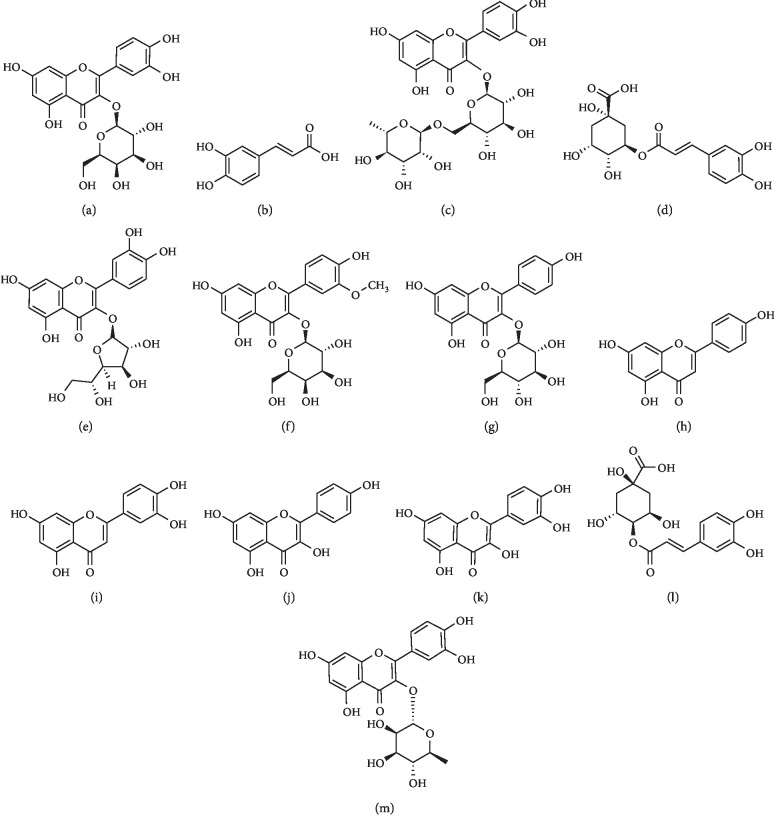
Chemical structures of 13 components. (a) Hyperoside. (b) Caffeic acid. (c) Rutin. (d) Chlorogenic acid. (e) Isoquercitrin. (f) Isorhamnetin-3-O-glucoside. (g) Astragalin. (h) Apigenin. (i) Luteolin. (j) Kaempferol (k) Quercetin. (l) Cryptochlorogenic acid. (m) Quercitrin.

Methanol and acetonitrile (HPLC pure grade) were purchased from Fisher Co., Ltd. Formic acid was of chromatographic purity obtained from ROE Co., Ltd. Ultrapure water for the HPLC-MS/MS analysis was purified by Milli-Q water purification system (Millipore, Milford, MA, USA). Hyperoside, caffeic acid, rutin, chlorogenic acid, luteolin, apigenin, kaempferol, isoquercitrin, cryptochlorogenic acid, isorhamnetin-3-*O*-glucoside, astragalin, and liquiritin (internal standards, IS) were obtained from Chengdu Must Bio-Technology Co., Ltd (Chengdu, China). Quercitrin and quercetin were purchased from National Institutes for Food and Drug Control. *Semen Cuscutae* was purchased from Anguo, Hebei province. The structures of 13 compounds are displayed in Figure 1.

Sprague–Dawley rats (SPF, 200 ± 10 g, male) were purchased from HFK Laboratory Animal Technology Co., Ltd (license number: SCXK 2014-0004).

### 2.2. Instruments and Experimental Conditions

The electrospray ionization (ESI) source was used to connect the HPLC-MS/MS system consisting of an Agilent 1200 high-performance liquid chromatography equipped with an Agilent 6430 series triple quadrupole mass spectrometer (Agilent Technologies, USA). The target compounds and IS were separated on the CORTECS®C_18_ column (4.6 × 150 mm, 2.7 *μ*m) with mobile phases consisting of 0.1% formic acid in water (A) and acetonitrile (B) at a flow rate of 0.3 mL/min. The gradient elution method as follows: 0–5 min, 25%–75% B; 5–7 min, 75%–95% B; 7–12 min, 95%–95% B. And the column balance procedure was 12–13 min, 95%–25% B; 13–17 min, 25%–25% B. The column temperature was maintained at 30°C and the injection volume set at 5 *μ*L. The data obtained were processed using Mass Hunter workstation software (Agilent Technologies, USA).

Quantification was achieved in the negative multiple reaction monitoring mode (MRM). Parameters were set as follows: capillary temperature 250°C, drying gas flow 9 L/min, and nebulizing gas pressure at 20 psi. The parameters in mass spectrometry analysis were set as listed in Table 1.

### 2.3. *Semen Cuscutae* Extract Preparation

3 kg of *Semen Cuscutae* were weighed accurately, crushed and sifted with No. 4 sieve, and extracted three times using continuous reflux method by 80% ethanol (v/v) in the volume of 15 L, 12 L, 10 L for 2 h, 1.5 h, and 1 h, respectively. The extraction was then filtered and mixed. The ethanol mixture was concentrated with reduced pressure and dried by vacuum to obtain the *Semen Cuscutae* extract. The herb extract was crushed into powder form and stored in a desiccator until analysis. The extract contains quercitrin, quercetin, hyperoside, caffeic acid, rutin, chlorogenic acid, luteolin, apigenin, kaempferol, isoquercitrin, cryptochlorogenic acid, isorhamnetin-3-*O*-glucoside, and astragalin 0.2, 88.9, 841.6, 69.1, 4.1, 452.8, 7.4, 1.0, 351.3, 834.8, 47.3, 168.8, and 461.7 *μ*g/g, respectively.

### 2.4. Preparation of Calibration Standards, QC, and IS Solutions

Stock solutions of quercitrin, quercetin, hyperoside, caffeic acid, rutin, chlorogenic acid, luteolin, apigenin, kaempferol, isoquercitrin, cryptochlorogenic acid, isorhamnetin-3-*O*-glucoside, astragalin, and liquiritin (IS) were prepared in methanol at 100 *μ*g/mL. The mixed stock solutions were further obtained by mixing the stock samples together and diluted with an appropriate volume of methanol.

The calibration curve of quercitrin, quercetin, hyperoside, caffeic acid, chlorogenic acid, luteolin, apigenin, kaempferol, isoquercitrin, cryptochlorogenic acid, isorhamnetin-3-*O*-glucoside, astragalin (1, 2, 5, 10, 25, 50, 100, 250 ng/mL), and rutin (2, 4, 10, 20, 50, 100, 200, 500 ng/mL) were prepared by adding the respective mixed stock solutions and 20 *μ*L of liquiritin (IS, 200 ng/mL) into 100 *μ*L blank plasma.

Quality control (QC) samples included low, middle, and high concentrations, prepared with the appropriate mixed stock solutions and blank plasma sample to meet the desired concentration. All the solutions were stored at −4°C.

### 2.5. Treatment of Plasma

20 *μ*L of methanol, 20 *μ*L of IS (liquiritin, 200 ng/mL) and 20 *μ*L of formic acid were added to 100 *μ*L of plasma sample and then vortex-mixed. The mixture was extracted with 800 *μ*L of acetonitrile by vortexing for 3 min. After centrifugation at 14000 rpm for 10 min, the supernatant was transferred to a clean glass tube and dried with nitrogen. The residue was reconstituted in 100 *μ*L of methanol and then centrifuged at 14000 rpm for 10 min. 5 *μ*L of supernatant was injected into the LC–MS/MS system for analysis.

### 2.6. Method Validation

Specificity, linearity, lower limit of quantitation (LLOQ), precision, accuracy, extraction recovery, matrix effect, and stability for the method were validated based on the guidelines published by regulatory authorities [15].

#### 2.6.1. Specificity

The specificity was investigated by analyzing the chromatography of blank plasma samples from six different rats to determine whether the endogenous substances in the sample would affect the quantitative analysis of each component.

#### 2.6.2. Linearity and LLOQ

The linearity was achieved by spiking rat plasma with the mixed standard solution and IS in a series of concentrations. The calibration curves were constructed with peak-area ratio of analyte to IS (*y*) against concentration of the calibration standard (*x*), with 1/*x*^2^ as the weighing factor. LLOQ was evaluated according to the lowest concentration of standard curve at which the signal-to-noise ratio (S : N) was about 10 : 1.

#### 2.6.3. Precision and Accuracy

Intra- and interday precision were obtained by determining QC samples at three concentration levels, i.e., low, middle, and high (consisting of 2, 25, and 250 ng/mL of hyperoside, caffeic acid, chlorogenic acid, luteolin, apigenin, kaempferol, isoquercitrin, cryptochlorogenic acid, isorhamnetin-3-*O*-glucoside, astragalin, quercitrin, quercetin, and 4, 50, and 500 ng/mL for rutin, respectively). All plasma samples were performed in six replicates at three concentrations. Relative standard deviations were used to determine precision, and accuracy was evaluated by RE.

#### 2.6.4. Matrix Effect and Extraction Recovery

The extraction recovery was assessed by comparing the peak areas obtained from pretreatment procedures samples with those from postextracted spiked samples. The matrix effect of the 13 analytes was determined by comparing the peak area obtained from postextracted spiked samples to that from pure standards solutions. All the extraction recovery and matrix effect experiments were evaluated in six replicates with three concentration levels.

#### 2.6.5. Stability

The stability of the 13 analytes was evaluated by QC samples in different processing and storage conditions, including short-term stability (putting the analytes in ambient temperature for 4 h and storing analytes in autosampler for 12 h after treatment), long-term stability (storing the analytes at −70°C for 21 days) and subjecting analytes to three freeze-thaw cycles. All stability experiments were tested in six parallels with low, middle, and high concentrations.

### 2.7. Pharmacokinetic Studies

Six Sprague–Dawley rats (SPF, 200 ± 10 g, male) were purchased from HFK Laboratory Animal Technology Co., Ltd. The rats were housed in a standard laboratory condition (12 h dark-light cycle; temperature was 25°C ± 2°C and humidity was kept 50 ± 5%) and fed standard dry pellet diet and water for one week for acclimatization. Before the experiments, the rats were in fasting state for 12 h, with free access to water. The *Semen Cuscutae* extracts were dissolved in 0.5% carboxymethyl cellulose-sodium, prepared into suspension and gavage to the rats at a dose of 13 g/kg. Approximately 200 *μ*L of rat blood samples was collected from the orbital venous plexus at 0, 0.03, 0.08, 0.17, 0.25, 0.5, 1, 2, 4, 6, 8, 10, 12, 24, 36, and 48 h after oral administration into 1.5 mL heparinized centrifuge tube. After centrifugation at 7000 rpm for 10 min immediately, the supernatant was collected into a new centrifuge tube and stored at −70°C until analysis.

### 2.8. Data Analysis

Pharmacokinetic parameters (*C*_max_, *t*_1/2_, *T*_max_, *AUC*_(0-*tn*)_, and *AUC*_(0-*∞*)_) were obtained using the software “Drug and Statistics 3.0” (DAS 3.0) (Medical College of Wannan, China).

## 3. Results and Discussion

### 3.1. Method Development

In order to improve sensitivity and shorten analysis time, we tested different columns and various mobile phase systems. Comparing different columns such as CORTECS®C_18_ column (4.6 mm × 150 mm, 2.7 *μ*m), Xbridge™C_18_ column (2.1 mm × 150 mm, 3.5 *μ*m), and Xbridge™C_18_ column (4.6 mm × 50 mm, 2.5 *μ*m), and various mobile phase systems such as acetonitrile-water or 0.1% formic acid in water and methanol-water or 0.1% formic acid in water, we found that the sensitivity and signal response of compounds analyzed with CORTECS®C_18_ column (4.6 mm × 150 mm, 2.7 *μ*m) and acetonitrile-water containing 0.1% formic acid were more satisfactory.

The standard solutions of the target compounds and IS were infused into the instrument separately, both positive and negative ion modes were compared to optimize mass conditions. The results showed that all analytes were better eluted under negative ionization mode. Optimized precursor-to-production transitions were observed at 447.0 ⟶ 299.9 for quercitrin, 300.9 ⟶ 151.0 for quercetin, 463.1 ⟶ 300.0 for hyperoside, 179.1 ⟶ 135.0 for caffeic acid, 609.2 ⟶ 300.1 for rutin, 353.0 ⟶ 191.3 for chlorogenic acid, 285.1 ⟶ 132.8 for luteolin, 269.0 ⟶ 117.0 for apigenin, 285.1 ⟶ 187.1 for kaempferol, 462.9 ⟶ 300.0 for isoquercitrin, 353.1 ⟶ 173.2 for cryptochlorogenic acid, 476.9 ⟶ 313.8 for isorhamnetin-3-*O*-glucoside, 447.1 ⟶ 284.1 for astragalin, and 416.9 ⟶ 255.0 for liquiritin (IS).

### 3.2. Sample Preparation

In our study, three methods were attempted at disposing the plasma sample, namely, liquid-liquid extraction (LLE) with ethyl acetate, protein precipitation (PPT) with acetonitrile, and protein precipitation (PPT) with methyl alcohol. The results showed that the method of PPT with acetonitrile showed higher extraction efficiency, lower matrix effect, and simpler operational flow. Meeting the requirements of this experiment in determining biological samples, protein precipitation (PPT) with acetonitrile was employed in this study for sample preparation.

### 3.3. Method Validation

#### 3.3.1. Specificity

**Figure 2:**
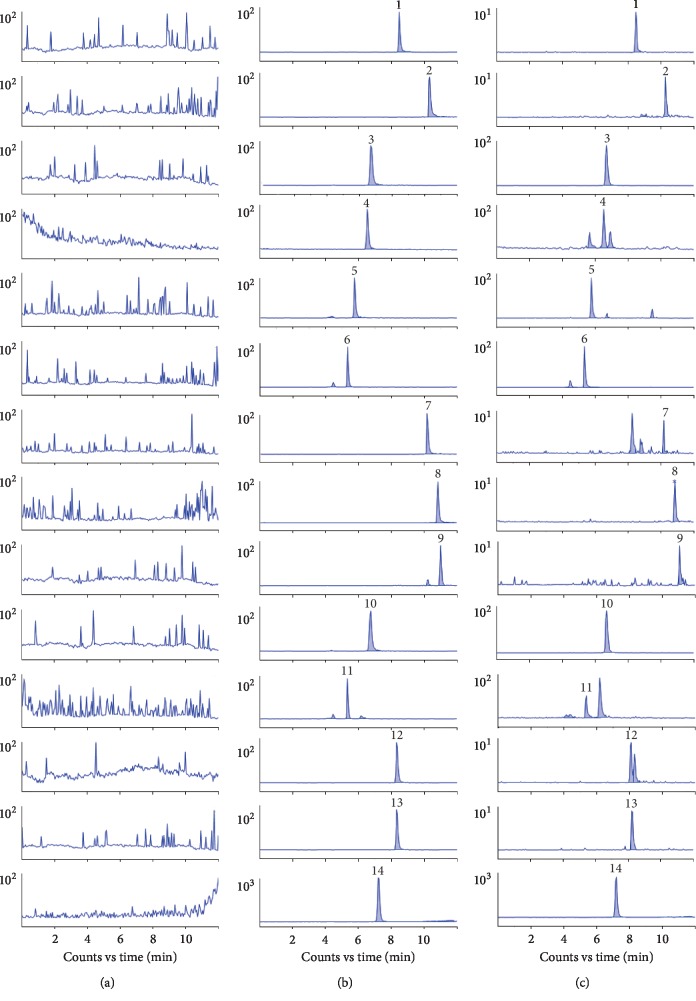
MRM chromatograms of 13 analytes. Quercitrin (1). Quercetin (2). Hyperoside (3). Caffeic acid (4). Rutin (5). Chlorogenic Acid (6). Luteolin (7). Apigenin (8). Kaempferol (9). Isoquercitrin (10). Cryptochlorogenic Acid (11). Isorhamnetin-3-O-glucoside (12). Astragalin (13). IS (14). (a) Blank plasma; (b) blank plasma spiked with the analytes and IS; (c) plasma sample collected at 0.25 h after oral administration of *Semen Cuscutae* extract.

Specificity was evaluated by testing blank plasma, plasma samples with target compounds, and plasma samples after oral administration of *Semen Cuscutae* extract from six different rats. The retention time of quercitrin, quercetin, hyperoside, caffeic acid, rutin, chlorogenic acid, luteolin, apigenin, kaempferol, isoquercitrin, cryptochlorogenic acid, isorhamnetin-3-*O*-glucoside, astragalin, and liquiritin (IS) were 8.53, 10.33, 6.73, 6.57, 5.81, 5.33, 10.23, 10.85, 10.99, 6.68, 5.32, 8.36, 8.22, and 7.19 min, respectively. The chromatograms as shown in Figure 2 suggested no interfering peaks from the endogenous matrix in rat blood sample.

#### 3.3.2. Linearity and Sensitivity

The data of the linear equation, correlation coefficients, linearity ranges, and LLOQ of all target compounds are listed in Table 2. The correlation coefficients of all analytes were greater than 0.9903, indicating that the 13 analytes in the plasma sample had good linearity in the corresponding concentration range. LLOQ with S/N ratio >10 ranged from 1–2 ng/mL of the 13 analytes, showing that the above method developed is suitable for quantitative pharmacokinetic studies.

#### 3.3.3. Precision and Accuracy

In this experiment, all results of the intra- and interday precision and accuracy were analyzed at three different concentration levels, including low, medium, and high concentrations in six replicates, as displayed in Table 3. Intra- and interday RSD for the analytes ranged from 0.5%–10.4% and 0.8–7.9%, respectively. RE of accuracy did not exceed ±14.5% for all analytes. The results implied that this method is reliable and accurate for the study of the above target compounds in rat plasma.

#### 3.3.4. Extraction Recovery and Matrix Effect

The results of extraction recovery and matrix effect are given in Table 4. The extraction recoveries of the analytes in rat plasma sample at three concentration levels ranged from 87.4% to 106.2%. The matrix effects of the target compounds were in the range of 81.0–115.5%. The data showed that the process of the experiment is efficient and there was no significant matrix effect observed for the plasma sample tested.

#### 3.3.5. Stability

Stability data of the 13 analytes in different conditions are listed in Table 5. All analytes subjected to different processing and storage conditions had an acceptance criterion in the range of 1.1%–11.1% for QC sample at three concentration levels. The results suggested that the analytes had a satisfactory stability for storage and analytical process.

### 3.4. Pharmacokinetic Application

**Figure 3:**
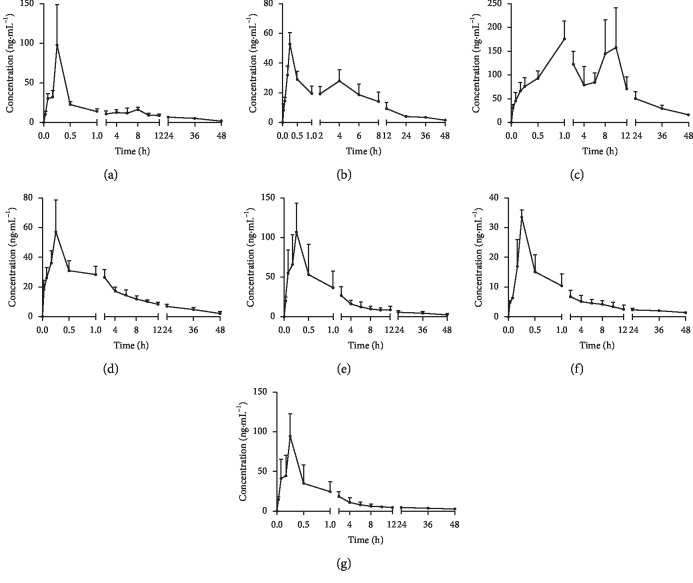
Mean plasma concentration-time curves of hyperoside, caffeic acid, rutin, chlorogenic acid, isoquercitrin, isorhamnetin-3-O-glucoside, and astragalin after oral administration of *Semen Cuscutae* extract at a single dose of 13 g/kg to SD rats (mean ± SD, *n* = 6). (a) Hyperoside. (b) Caffeic acid. (c) Rutin. (d) Chlorogenic acid. (e) Isoquercitrin. (f) Isorhamnetin-3-O-glucoside. (g) Astragalin.

The validated method was successfully applied to a pharmacokinetic study of orally administered *Semen Cuscutae* extract (13.0 g/kg) in rats with the determination of 13 active ingredients of *Semen Cuscutae* in rat plasma. The corresponding pharmacokinetic parameters are listed in Table 6. Mean plasma concentration-time profiles of target compounds were illustrated in Figure 3.

In our experiment, 6 ingredients, namely, quercitrin, quercetin, apigenin, kaempferol, luteolin, and cryptochlorogenic acid were found to be of low content in vivo, and their concentrations after 15 minutes of intragastric administration were lower than the LLOQ. Therefore, complete pharmacokinetic curves of the above 6 compounds were not obtained. In the previous studies, researchers have explored the pharmacokinetics rules of kaempferol and quercetin [16, 17]. Compared with our study, the dose of oral administration was significantly higher than ours, and caused by the low oral bioavailability of them, we could just detect them at a few time points, and a whole mean plasma concentration-time curve could not be obtained. Hence, their pharmacokinetic parameters were not further discussed.

The *T*_max_ of hyperoside, chlorogenic acid, isoquercitrin, isorhamnetin-3-*O*-glucoside, and astragalin was less than 1 h. These results showed that the 5 ingredients were absorbed quickly in vivo. The elimination half-life (*t*_1/2_) of caffeic acid and rutin was more than 7.5 h, suggesting that caffeic acid and rutin are present in the body for a longer time, and may exert continuous therapeutic action, enhancing clinical efficacy. Similar pharmacokinetic trends have been reported in previous studies [18, 19]. In addition to this, the *t*_1/2_ of hyperoside, astragalin, isoquercitrin, chlorogenic acid, and sorhamnetin-3-*O*-glucoside was less than 1.5 h, indicating that the above compounds were eliminated quickly after intragastric administration of *Semen Cuscutae* extract.

In the previous study of our research group, we have established a HPLC-MS/MS method to explore the pharmacokinetic rule of rutin from mulberry leaves [16]. The results were different from this study. In this research, we found that rutin had an isomeride from *Semen Cuscutae* extract, that is, the isomeride with a high concentration. However, its plasma concentration was low. So, we guessed this isomeride may transform into rutin after oral administration in vivo. Thus, in the pharmacokinetic application, *C*_max_ of rutin is higher than that of other components.

## 4. Conclusions

A validated and selective method of HPLC-MS/MS for simultaneous quantification of 13 compounds of *Semen Cuscutae* was established in this study, and the pharmacokinetics of *Semen Cuscutae* extract in rats was investigated. We found that the content of quercitrin, quercetin, apigenin, kaempferol, luteolin, and cryptochlorogenic acid were at a lower level in vivo after oral administration, which could only be detected at a few time points. Meanwhile, other compounds except caffeic acid and rutin had shorter elimination half-life. The pharmacokinetic parameters indicate the metabolism rate of these elements and may provide references for further research of *Semen Cuscutae*.

## Figures and Tables

**Table 1:** The mass spectrometry of 13 compounds and IS.

Compound	Ion masses	Formula	Retention time	Precursor ion	Product ion	Fragment ions	CE (V)
Caffeic acid	180.157	C_9_H_8_O_4_	6.57	179.1	135.0	135.0	12
Apigenin	270.237	C_15_H_10_O_5_	10.85	269.0	117.0	116.9, 151.2	34
Luteolin	286.236	C_15_H_10_O_6_	10.23	285.1	132.8	132.8	29
Kaempferol	286.236	C_15_H_10_O_6_	10.99	285.1	187.1	93.1, 117.2, 205.0	25
Quercetin	302.236	C_15_H_10_O_7_	10.33	300.9	151.0	197.8, 179.1, 151.0	15
Cryptochlorogenic acid	354.309	C_16_H_18_O_9_	5.33	353.1	173.2	191.0	11
Chlorogenic acid	354.309	C_16_H_18_O_9_	5.32	353.0	191.3	191.3	11
Quercitrin	448.377	C_21_H_20_O_11_	8.53	447.0	299.9	299.9	22
Astragalin	448.377	C_21_H_20_O_11_	8.22	477.1	284.1	284.1	25
Isoquercitrin	464.376	C_21_H_20_O_12_	6.68	462.9	300.0	300.0	24
Hyperoside	464.376	C_21_H_20_O_12_	6.73	463.1	300.0	300.0, 271.2	31
Isorhamnetin-3-O-glucoside	478.403	C_22_H_22_O_12_	8.36	476.9	313.8	300.9, 178.9, 150.9	10
Rutin	610.518	C_27_H_30_O_16_	5.81	609.2	300.1	300.1, 272.3	34
Liquiritin (IS)	418.39	C_21_H_22_O_9_	7.19	416.9	255.0	179.0	17

**Table 2:** Calibration curves, correlation coefficients, linear ranges, and LLOQ of the analytes.

Compound	Calibration curves	Correlation coefficients (r)	Linear range (ng/mL)	LLOQ (ng/mL)
Quercitrin	*Y* = 0.6491*X* + 0.0086	0.9925	1–250	1
Quercetin	*Y* = 0.5980*X* + 0.0026	0.9967	1–250	1
Hyperoside	*Y* = 0.8758*X* + 0.0098	0.9961	1–250	1
Caffeic acid	*Y* = 0.8239*X* + 0.0030	0.9986	1–250	1
Rutin	*Y* = 0.2459*X* + 0.0138	0.9920	2–500	2
Chlorogenic acid	*Y* = 0.1467*X* + 0.0038	0.9914	1–250	1
Luteolin	*Y* = 0.6678*X* + 0.0025	0.9936	1–250	1
Apigenin	*Y* = 0.7207*X* + 0.0017	0.9929	1–250	1
Kaempferol	*Y* = 0.0250*X* − 0.0001	0.9952	1–250	1
Isoquercitrin	*Y* = 0.8758*X* + 0.0098	0.9960	1–250	1
Cryptochlorogenic acid	*Y* = 0.1038*X* + 0.0003	0.9903	1–250	1
Isorhamnetin-3-O-glucoside	*Y* = 0.5362*X* + 0.0036	0.9942	1–250	1
Astragalin	*Y* = 0.6307*X* + 0.0052	0.9962	1–250	1

**Table 3:** Precision and accuracy of 13 analytes in rat plasma (*n* = 6).

Compounds	Spiked concentration (ng/mL)	Intraday			Interday		
		Measured concentration (ng mL^−1^)	Accuracy (RE, %)	Precision (RSD, %)	Measured concentration (ng mL^−1^)	Accuracy (RE, %)	Precision (RSD, %)
Quercitrin	2	1.94 ± 0.05	−3.0	2.6	1.95 ± 0.05	−2.5	2.6
	25	27.65 ± 1.75	10.6	6.3	26.16 ± 0.94	4.6	3.6
	250	253.47 ± 4.06	1.4	1.6	264.45 ± 10.46	5.8	4.0
	2	1.99 ± 0.10	−0.5	5.0	2.02 ± 0.04	1.0	2.0
Quercetin	25	26.60 ± 1.02	6.4	3.8	25.56 ± 0.68	2.2	2.7
	250	262.38 ± 5.97	5.0	2.3	252.55 ± 3.26	1.0	1.3
	2	1.98 ± 0.12	−1.0	6.1	2.05 ± 0.05	2.5	2.4
Hyperoside	25	28.58 ± 0.33	14.3	1.2	28.21 ± 0.39	12.8	1.4
	250	277.08 ± 12.08	10.8	4.4	270.47 ± 4.99	8.2	1.8
Caffeic acid	2	2.14 ± 0.06	7.0	2.8	2.00 ± 0.06	−0.1	3.0
	25	25.92 ± 1.78	3.7	6.9	25.83 ± 0.47	3.3	1.8
	250	257.37 ± 5.90	2.9	2.3	250.56 ± 2.29	0.2	0.9
Rutin	4	4.24 ± 0.17	6.0	4.0	4.16 ± 0.13	4.0	3.1
	50	55.09 ± 2.26	10.2	4.1	49.67 ± 1.34	−0.7	2.7
	500	494.24 ± 8.21	−1.2	1.7	498.04 ± 5.47	−0.4	1.1
Chlorogenic acid	2	1.98 ± 0.03	−1.0	1.5	1.90 ± 0.15	−5.0	7.9
	25	26.10 ± 2.72	4.4	10.4	26.28 ± 0.73	5.1	2.8
	250	254.48 ± 7.30	1.8	2.9	254.24 ± 4.74	1.7	1.9
	2	2.18 ± 0.14	9.0	6.4	1.96 ± 0.05	−2.0	2.6
Luteolin	25	26.98 ± 1.74	7.9	6.4	25.97 ± 1.16	3.9	4.5
	250	256.22 ± 6.42	2.5	2.5	255.18 ± 3.16	2.1	1.2
Apigenin	2	2.12 ± 0.14	6.0	6.6	2.03 ± 0.09	1.5	4.4
	25	27.22 ± 1.31	8.9	4.8	25.60 ± 0.48	2.4	1.9
	250	258.13 ± 10.48	3.3	4.1	261.87 ± 13.20	4.7	5.0
Kaempferol	2	2.03 ± 0.06	1.5	3.0	2.05 ± 0.10	2.5	4.9
	25	25.92 ± 0.84	3.7	3.2	25.43 ± 0.58	1.7	2.3
	250	253.01 ± 7.68	1.2	3.0	266.49 ± 14.27	6.6	5.4
Isoquercitrin	2	2.02 ± 0.07	1.0	3.5	2.00 ± 0.07	0.1	3.5
	25	28.62 ± 0.62	14.5	2.2	28.29 ± 1.06	13.2	3.7
	250	272.50 ± 4.96	9.0	1.8	273.47 ± 8.62	9.4	3.2
Cryptochlorogenic acid	2	2.03 ± 0.06	1.5	3.0	2.00 ± 0.08	0.1	4.0
	25	25.92 ± 0.84	3.7	3.2	25.61 ± 1.03	2.4	4.0
	250	253.01 ± 7.68	1.2	3.0	251.30 ± 1.99	0.5	0.8
Isorhamnetin-3-O-glucoside	2	1.99 ± 0.07	−0.5	3.5	2.02 ± 0.05	1.0	2.5
	25	26.18 ± 0.85	4.7	3.2	25.53 ± 0.58	2.1	2.3
	250	254.51 ± 1.60	1.8	0.6	250.00 ± 3.23	0.0	1.3
Astragalin	2	1.95 ± 0.05	−2.5	2.6	2.00 ± 0.04	−0.1	2.0
	25	26.19 ± 0.93	4.8	3.6	25.07 ± 0.39	0.3	1.6
	250	248.93 ± 1.24	−0.4	0.5	253.07 ± 2.55	1.2	1.0

**Table 4:** Extraction recoveries and matrix effects of the analytes (*n* = 6).

Compounds	Concentration (ng/mL)	Extraction recovery (%)	RSD (%)	Matrix effect (%)	RSD (%)
Quercitrin	2	97.3 ± 5.1	5.2	105.8 ± 4.8	4.5
	25	97.5 ± 2.8	2.9	102.1 ± 2.4	2.4
	250	87.7 ± 2.0	2.3	106.7 ± 4.3	4.0
Quercetin	2	89.9 ± 7.3	8.1	115.5 ± 11.6	10.0
	25	98.7 ± 3.8	3.9	93.5 ± 7.2	7.7
	250	87.7 ± 1.8	2.1	97.0 ± 4.3	4.4
Hyperoside	2	103.9 ± 4.4	4.2	101.4 ± 6.2	6.1
	25	102.3 ± 6.3	6.2	97.1 ± 5.7	5.9
	250	101.7 ± 0.6	0.6	81.1 ± 2.9	3.6
Caffeic acid	2	98.8 ± 7.1	7.2	101.0 ± 4.5	4.5
	25	102.0 ± 2.9	2.8	86.9 ± 11.2	12.9
	250	96.2 ± 4.8	5.0	87.4 ± 3.0	3.4
Rutin	4	103.2 ± 4.6	4.5	103.3 ± 3.9	3.8
	50	92.0 ± 3.7	4.0	95.3 ± 7.2	7.6
	500	90.7 ± 5.2	5.7	85.9 ± 10.7	12.5
Chlorogenic acid	2	103.6 ± 5.1	4.9	97.9 ± 9.1	9.3
	25	97.8 ± 2.0	2.0	98.2 ± 6.1	6.2
	250	95.4 ± 1.3	1.4	85.1 ± 6.4	7.5
Luteolin	2	106.2 ± 4.5	4.2	95.3 ± 6.0	6.3
	25	95.3 ± 3.5	3.7	103.9 ± 9.9	9.5
	250	87.4 ± 2.6	3.0	97.4 ± 2.4	2.5
Apigenin	2	99.1 ± 6.7	6.8	95.4 ± 4.5	4.7
	25	91.4 ± 2.6	2.8	81.0 ± 2.3	2.8
	250	88.5 ± 1.5	1.7	88.7 ± 2.1	2.4
Kaempferol	2	99.1 ± 5.2	5.2	105.6 ± 15.2	14.4
	25	94.7 ± 4.2	4.4	96.6 ± 3.8	3.9
	250	91.4 ± 1.7	1.9	85.9 ± 1.0	1.2
Isoquercitrin	2	104.3 ± 2.2	2.1	97.3 ± 2.7	2.8
	25	104.7 ± 6.7	6.4	93.9 ± 2.3	2.4
	250	101.8 ± 2.6	2.6	83.2 ± 2.1	2.5
Cryptochlorogenic acid	2	94.7 ± 4.9	5.2	94.5 ± 7.6	8.0
	25	94.0 ± 3.6	3.8	111.2 ± 1.7	1.5
	250	90.8 ± 3.1	3.4	81.9 ± 3.1	3.8
Isorhamnetin-3-O-glucoside	2	99.3 ± 5.4	5.4	109.9 ± 3.8	3.5
	25	96.4 ± 5.2	5.4	99.8 ± 5.0	5.0
	250	88.8 ± 2.5	2.8	102.3 ± 3.4	3.3
Astragalin	2	97.9 ± 5.1	5.2	115.1 ± 4.9	4.3
	25	98.7 ± 3.9	4.0	98.7 ± 1.2	1.2
	250	87.6 ± 3.9	4.5	103.8 ± 4.9	4.7

**Table 5:** Stability of all analytes in rat plasma (*n* = 6).

Compounds	Spiked concentration (ng/mL)	Room temperature for 4h		Three freeze-thaw		Autosampler for 12h		−70°C for 21 days	
		Measured (ng/mL)	RSD (%)	Measured (ng/mL)	RSD (%)	Measured (ng/mL)	RSD (%)	Measured (ng/mL)	RSD (%)
Quercitrin	2	2.1 ± 0.1	4.8	2.3 ± 0.1	4.3	2.1 ± 0.1	4.8	2.0 ± 0.1	5.0
	25	26.3 ± 0.7	2.7	26.9 ± 1.5	5.6	26.4 ± 0.9	3.4	26.5 ± 0.5	1.9
	250	252.5 ± 9.1	3.6	261.4 ± 10.3	3.9	253.1 ± 4.7	1.9	247.9 ± 5.3	2.1
Quercetin	2	2.2 ± 0.1	4.5	2.0 ± 0.1	5.0	2.3 ± 0.1	4.3	1.9 ± 0.1	5.3
	25	26.2 ± 0.7	2.7	27.3 ± 0.6	2.2	26.6 ± 0.7	2.6	25.6 ± 0.9	3.5
	250	254.9 ± 11.8	4.6	253.2 ± 5.0	2.0	245.3 ± 11.6	4.7	260.0 ± 6.2	2.4
Hyperoside	2	2.0 ± 0.1	5.0	2.1 ± 0.1	4.8	1.8 ± 0.1	5.6	1.9 ± 0.1	5.3
	25	23.0 ± 1.2	5.2	26.2 ± 1.5	5.7	23.6 ± 0.7	3.0	26.4 ± 1.8	6.8
	250	244.4 ± 14.3	5.9	254.7 ± 9.7	3.8	230.2 ± 13.1	5.7	256.4 ± 15.8	6.2
Caffeic acid	2	2.1 ± 0.1	4.8	2.1 ± 0.1	4.8	2.2 ± 0.1	4.5	2.0 ± 0.1	5.0
	25	27.9 ± 1.0	3.6	27.0 ± 1.3	4.8	28.1 ± 0.8	2.8	26.0 ± 0.6	2.3
	250	272.5 ± 8.9	3.3	255.7 ± 3.9	1.5	262.6 ± 7.4	2.8	250.2 ± 5.1	2.0
Rutin	4	3.9 ± 0.2	5.1	4.0 ± 0.1	2.5	4.0 ± 0.2	5.0	4.0 ± 0.1	2.5
	50	56.3 ± 2.3	4.1	57.7 ± 1.0	1.7	49.8 ± 2.7	5.4	52.7 ± 3.9	7.4
	500	505.7 ± 13.0	2.6	513.1 ± 7.4	1.4	489.3 ± 14.2	2.9	494.4 ± 8.8	1.8
Chlorogenic acid	2	2.1 ± 0.1	4.8	2.1 ± 0.1	4.8	2.1 ± 0.1	4.8	2.1 ± 0.1	4.8
	25	27.7 ± 1.6	5.8	28.1 ± 1.0	3.6	26.6 ± 1.6	6.0	26.6 ± 1.2	4.5
	250	258.4 ± 15.7	6.1	268.6 ± 15.8	5.9	253.1 ± 6.0	2.4	253.6 ± 7.7	3.0
Luteolin	2	2.2 ± 0.1	4.5	2.0 ± 0.1	5.0	2.1 ± 0.1	4.8	2.0 ± 0.1	5.0
	25	26.5 ± 0.5	1.9	26.8 ± 1.6	6.0	25.6 ± 1.0	3.9	27.4 ± 0.9	3.3
	250	256.7 ± 11.5	4.5	261.1 ± 8.2	3.1	244.9 ± 6.3	2.6	257.6 ± 5.1	2.0
Apigenin	2	2.2 ± 0.1	4.5	2.0 ± 0.1	5.0	2.1 ± 0.2	9.5	2.1 ± 0.1	4.8
	25	26.4 ± 1.2	4.5	25.7 ± 2.0	7.8	25.0 ± 1.2	4.8	26.5 ± 1.3	4.9
	250	249.3 ± 12.8	5.1	262.2 ± 16.0	6.1	248.5 ± 4.9	2.0	257.8 ± 8.2	3.2
Kaempferol	2	2.2 ± 0.2	9.1	2.0 ± 0.2	10.0	2.1 ± 0.2	9.5	2.0 ± 0.1	5.0
	25	24.8 ± 0.3	1.2	26.2 ± 1.6	6.1	25.4 ± 2.5	9.8	25.7 ± 0.8	3.1
	250	247.5 ± 6.3	2.5	252.6 ± 7.5	3.0	254.0 ± 4.9	1.9	257.2 ± 6.4	2.5
Isoquercitrin	2	1.8 ± 0.2	11.1	2.1 ± 0.1	4.8	1.9 ± 0.1	5.3	1.9 ± 0.1	5.3
	25	22.8 ± 1.4	6.1	26.2 ± 1.8	6.9	24.0 ± 0.5	2.1	25.9 ± 1.8	6.9
	250	247.2 ± 14.7	5.9	248.9 ± 5.1	2.0	238.3 ± 7.2	3.0	263.6 ± 10.2	3.9
Cryptochlorogenic acid	2	2.0 ± 0.1	5.0	2.0 ± 0.1	5.0	2.1 ± 0.1	4.8	2.0 ± 0.1	5.0
	25	27.0 ± 1.8	6.7	26.2 ± 0.3	1.1	27.3 ± 0.6	2.2	25.5 ± 1.2	4.7
	250	260.4 ± 3.3	1.3	248.9 ± 6.5	2.6	251.2 ± 9.7	3.9	244.4 ± 7.4	3.0
Isorhamnetin-3-*O*-glucoside	2	2.0 ± 0.1	5.0	2.0 ± 0.1	5.0	2.1 ± 0.1	4.8	2.1 ± 0.1	4.8
	25	27.1 ± 1.3	4.8	27.1 ± 1.5	5.5	26.9 ± 1.0	3.7	26.2 ± 0.6	2.3
	250	259.9 ± 5.5	2.1	255.0 ± 5.7	2.2	251.5 ± 5.4	2.1	249.5 ± 7.0	2.8
Astragalin	2	2.1 ± 0.1	4.8	2.1 ± 0.1	4.8	2.1 ± 0.1	4.8	2.0 ± 0.1	5.0
	25	27.6 ± 1.2	4.3	27.2 ± 0.8	2.9	27.3 ± 1.0	3.7	26.1 ± 1.2	4.6
	250	248.1 ± 4.3	1.7	258.6 ± 8.5	3.3	254.6 ± 4.6	1.8	251.5 ± 8.7	3.5

**Table 6:** Pharmacokinetic parameters of 7 analytes after oral administration of *Semen Cuscutae* extract (*n* = 6).

Compounds	*T*_max_ (h)	*C*_max_ (ng/mL)	*t*_1/2_ (h)	AUC_(0-*tn*)_ (h·ng/mL)	AUC_(0-∞)_ (h·ng/mL)
Hyperoside	0.17 ± 0.07	29.81 ± 12.78	0.36 ± 0.23	684.47 ± 406.92	753.93 ± 423.71
Caffeic acid	0.28 ± 0.04	33.66 ± 8.43	11.60 ± 0.92	613.06 ± 290.85	651.94 ± 291.89
Rutin	10.00 ± 0.03	157.68 ± 84.08	7.70 ± 0.90	4463.92 ± 2274.52	4793.86 ± 2243.02
Chlorogenic acid	0.22 ± 0.06	35.51 ± 7.30	1.02 ± 0.67	994.54 ± 654.39	1000.96 ± 645.80
Isoquercitrin	0.21 ± 0.07	52.61 ± 7.55	0.49 ± 0.05	838.79 ± 518.04	949.49 ± 594.15
Isorhamnetin-3-*O*-glucoside	0.30 ± 0.01	19.51 ± 9.80	1.23 ± 0.26	171.70 ± 49.21	256.76 ± 74.37
Astragalin	0.24 ± 0.09	58.18 ± 28.53	0.45 ± 0.26	370.23 ± 258.63	440.81 ± 291.89

## Data Availability

The data used to support the findings of this study are available from the corresponding author upon request.
